# A Comparative Study of the Fatty Acid Profile of Non-Edible and Edible Tissues of Raw and Processed Common Octopus (*Octopus vulgaris*)

**DOI:** 10.3390/md23050182

**Published:** 2025-04-24

**Authors:** Luis Freiría-Martínez, Marcos Trigo, Ricardo Prego, Santiago P. Aubourg

**Affiliations:** 1 Department of Food Technology, Marine Research Institute (CSIC), c/E. Cabello 6, 36208 Vigo, Spain; lfreiria@iim.csic.es (L.F.-M.); mtrigo@iim.csic.es (M.T.); 2 Department of Oceanography, Marine Research Institute (CSIC), c/E. Cabello 6, 36208 Vigo, Spain; prego@iim.csic.es

**Keywords:** octopus, viscera, arm, mantle, cooking, frozen storage, ω3 fatty acids, ω3/ω6 ratio, EPA, DHA

## Abstract

A comparative study of the fatty acid (FA) composition of non-edible (viscera) and edible (mantle and arm) tissues of octopus (*Octopus vulgaris*) was carried out. According to the specimen size, three different groups (1–2 kg, 2–3 kg, and 3–4 kg, respectively) were taken into account. The effect of the cooking process (40 min at 90 °C) and frozen storage (4 months at −18 °C) was analyzed. In all kinds of samples, the polyunsaturated FA (PUFA) group was the most abundant (*p* < 0.05) and monounsaturated FAs were the least abundant (*p* < 0.05). Lower (*p* < 0.05) ω3-PUFA, ω3/ω6 ratio and docosahexaenoic acid values were detected in viscera (35.4–41.9%, 3.0–4.5%, and 12.7–17.5%, respectively) than in edible tissues (44.4–52.5%, 4.1–6.1%, and 24.3–30.1%, respectively). Conversely, higher (*p* < 0.05) eicosapentaenoic acid content was detected in viscera (19.6–21.9%) than in the edible tissues (17.2–19.3%). In most cases, the cooking process and frozen storage led to an average decrease in the PUFA and ω3-PUFA content and to an increase in the saturated FA presence. In agreement with current nutritional recommendations, all tissues showed great levels of highly valuable indices regarding the lipid fraction. The study proves that viscera, a waste substrate, can be considered a relevant source for food and pharmaceutical industrial requirements.

## 1. Introduction

Seafood is known to provide high contents of important constituents for the human diet. The great biological and chemical diversity in marine fish and invertebrate makes them a relevant source of highly valuable constituents susceptible to be used in a wide range of applications [1,2]. In agreement with a great number of studies focused on the employment of marine-enriched diets, the marine fatty acid (FA) profile has been found to be responsible for different health benefits (i.e., decreases in cardiovascular, neurological and inflammatory disorders) [3,4,5]. Notably, the consumption of eicosapentaenoic acid (EPA) and docosahexaenoic (DHA) acid consumption has been associated with low prevalence of different diseases related to neurodegenerative and cardiovascular problems [6,7].

The chemical composition of marine species has proved to encompass wide variations resulting from endogenous (genetic, anatomical, and physiological) and exogenous (water temperature, season, and feeding availability) factors [8]. Endogenous factors influence the distribution of biochemical constituents within different body tissues of marine species [9,10,11]. Among these constituents, lipid content and composition exhibit the greatest variability, differing significantly among edible tissues in wild invertebrates [12,13], fatty [14] and lean [15,16] fish species, and farmed fish [17,18].

The processing of marine species leads to a wide quantity of undesired by-products. In general, heads, blood, viscera, skin, or tails are obtained at different steps of seafood processing and constitute an important drawback for environmental contamination [19,20,21]. However, seafood by-products have been reported to be an important source of major components like proteins, minerals, lipids, and vitamins, in addition to minor constituents such as chitin, enzymes, pigments, and collagen [22,23,24]. Notably, the highest levels of high-added-value constituents is often present in marine tissues or parts that are often discarded [25,26].

Cephalopods are considered to be a highly interesting biological group because of their great nutritional value for human health and for their great commercial value [27,28,29]. Among cephalopods, the different species of octopus are considered as a highly nutritional seafood that can be commercialized in a wide range of products [30,31]. In the present research, a comparative study of FA composition was carried out between non-edible (viscera as a whole) and edible (mantle and arm) tissues of common octopus (*Octopus vulgaris*). The aim of the study was to prove the valuable FA composition of all tissues with a special stress on the viscera tissue, a waste product resulting from commercial processing. On the basis of the specimen sizes, three different groups were considered separately. Additionally, the effects on the FA profile of the cooking process and frozen storage were analysed.

## 2. Results and Discussion

### 2.1. FA Composition of Raw Samples: Effect of Tissue

The FA profiles of the different tissues were analyzed in the raw samples corresponding to the three sizes, i.e., Group I (1–2 kg per specimen; small-sized specimens), Group II (2–3 kg per specimen; medium-sized specimens), and Group III (3–4 kg per specimen; large-sized specimens). Throughout the whole study, each group was considered separately in order to carry out the comparison among tissues.

For the viscera tissue, the most abundant FA (g·100 g^−1^ total FAs) in all groups was EPA (19.5–22.0 range), followed by DHA (12.7–17.5 range), C16:0 (13.9–15.2 range), C18:0 (9.3–11.2 range), and C20:4ω6 (6.1–7.6 range). A previous seasonal study on several non-edible tissues of octopus (*O. vulgaris*) detected the same main FAs [32]; thus, the following decreasing sequences were detected for the digestive gland and the ovary: DHA > EPA > 16:0 > C18:0 > C20:4ω6 and DHA > 16:0 > EPA > C20:4ω6 > C18:0, respectively. The same main FAs (DHA, EPA, C16:0, C20:4ω6, and C18:0) than in the present study were also detected as the most abundant in octopus (*O. vulgaris*) by-products considered as a whole after lipid extraction by using low-toxicity solvents (acetone, ethanol, and ethyl acetate) [33].

Regarding the composition of non-edible tissues obtained from other cephalopod species, the same most abundant FAs (DHA, C16:0, and EPA) as in the current study were detected in total by-products obtained from Patagonian squid (*Doryteuthis gahi*) during a seasonal study [34]. However, C18:1ω9, DHA, and C20:1ω11 FAs were found to be the most abundant in cuttlefish (*Sepiella maindroni de Rochebrum*) viscera [35], and C16:0, EPA, and C18:0 FAs were observed as the most abundant in *Sepia officinalis* viscera (i.e., stomach, intestines, and pyloric caeca) [36]. A different distribution of the main FAs than in the present work was also detected by Singh et al. [28] in squid (*Loligo formasana*) ovary; in their study, DHA, EPA, and C20:4ω6 were found as the major FAs. Total viscera obtained from squid (*Illex argentinus*) showed the following decreasing sequence for the most abundant FAs: DHA > C16:0 > C18:1ω9 > EPA [37].

Regarding mantle and arm tissues in the present research, the most abundant FA (g·100 g^−1^ total FAs) in all groups was DHA (22.0–26.1 and 23.9–28.5 ranges, respectively), followed by C16:0 (16.8–17.0 and 14.3–19.1 ranges, respectively), EPA (16.7–18.6 and 17.2–19.0 ranges, respectively), C20:4ω6 (6.3–9.4 and 6.2–9.0 ranges, respectively), and C20:1ω9 (3.3–3.5 and 3.4–3.5 ranges, respectively). A similar FA distribution was detected in previous research related to the edible tissues of the current species of octopus. This distribution was observed in the mantle [32,38] and arm [32,39] tissues and in the edible tissues considered as a whole [40,41,42,43].

With the aim of better focusing on possible composition changes, discussion of FA values will be addressed in the present study to FA groups (saturated FAs, STFAs; monounsaturated FAs, MUFAs; PUFAs; ω3-PUFAs) and FA ratios (total ω3/total ω6; polyene index, PI; flesh-lipid quality, FLQ). Additionally, and on the basis of the importance of ω3-PUFAs, analysis of the content of single ω3-PUFAs (EPA; DHA; docosapentaenoic acid, DPA) will also be discussed.

#### 2.1.1. FA Groups

Values obtained for the STFA, MUFA, and PUFA groups in raw samples are shown in Table 1, Table 2 and Table 3, respectively. In all specimen sizes, the PUFA group showed to be the most abundant (ca. 47–62 g·100 g^−1^ total FAs range) and the MUFA group provided the lowest values (*p* < 0.05) (ca. 10–24 g·100 g^−1^ total FAs range).

Regarding the STFA group, no differences (*p* > 0.05) were detected among tissues for the small-sized specimens (Group I). Conversely, higher average values were detected in the mantle tissue than in the other tissues in the medium- and large-sized samples (Groups II and III, respectively); differences were found to be significant (*p* < 0.05) in both cases in comparison to the viscera tissue. A marked higher MUFA value (*p* < 0.05) was obtained in the viscera tissue when compared to the two other tissues; meanwhile, no differences (*p* > 0.05) were observed between mantle and arm tissues. In the case of the PUFA group, the opposite distribution as for the MUFA group was detected. Thus, the viscera tissue depicted the lowest values (*p* < 0.05) in all sizes (Groups I, II, and III). Comparisons of arm and mantle tissues revealed higher PUFA values (*p* < 0.05) in the mantle tissue for small-sized specimens; conversely, higher levels (*p* < 0.05) were detected in the arm tissue in the case of medium-sized octopus.

Regarding non-edible tissues, the same FA group distribution as in the current study was previously detected in octopus (*O. vulgaris*) ovary during a seasonal study carried out in specimens obtained in the Atlantic coast [32]; conversely, the digestive gland showed the following decreasing sequence: PUFAs > MUFAs = STFAs. During a recent study focused on lipid extraction with low-toxicity solvents [33], PUFAs were found to be the most abundant group, and MUFAs depicted the lowest values in total non-edible tissues from octopus (*O. vulgaris*). Also in agreement with the present study, the PUFAs > STFAs > MUFAs decreasing sequence was detected for FA groups in non-edible tissues obtained from Patagonian squid (*D. gahi*) [34] and viscera obtained from squid (*I. argentinus*) [37], cuttlefish (*S. officinalis*) [36], and Giant squid (*Dosidicus gigas*) [44].

A similar distribution of the three FA groups than in the present case was already detected in different edible tissues of octopus (*O. vulgaris*). This result has been observed in specimens also captured in the European Atlantic coast for mantle and arm tissues [32], and for edible parts considered as a whole [40,43]. Additionally, a similar FA distribution was also observed in specimens captured in the Mediterranean Sea for the mantle [38], arm [39], and edible parts [41], as well as in specimens obtained in the Brazilian coast [42].

**Figure 1:**
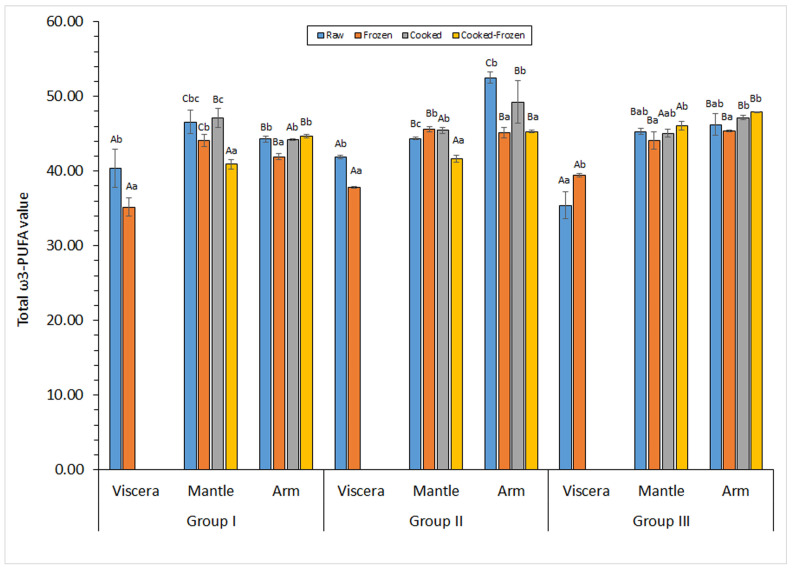
Determination of the total ω3 polyunsaturated fatty acid (ω3-PUFA) content (g·100 g^−1^ total FAs) in different kinds of raw and processed tissues corresponding to different specimen sizes. Mean values of three replicates (*n* = 3); standard deviations are indicated by bars. Within each group and for each raw/processed substrate, different capital letters (A,B,C) indicate significant differences (*p* < 0.05) among tissues. Within each group and for each tissue, different lowercase letters (a,b,c) indicate significant differences (*p* < 0.05) as a result of processing. Specimen groups are as expressed in Table 1. The viscera tissue was not subjected to the cooking process.

Marine lipids are reported to include many beneficial constituents for the human health, especially related to their high values on ω3-PUFAs [3,4]. On the basis of the great significance of ω3-PUFAs, its content was studied in the current research. Thus, a similar distribution was detected for the ω3-PUFAs (Figure 1) as for the PUFA group (Table 3). Values obtained in raw samples of the different size groups were included in the 35.4–41.9, 44.4–46.6, and 44.30–52.5 g·100 g^−1^ total FAs ranges for viscera, mantle and arm tissues, respectively (Figure 1). The lowest levels (*p* < 0.05) were detected in the viscera tissue of all sizes. Additionally, the mantle tissue showed higher values (*p* < 0.05) than the arm tissue for specimens of small sizes; conversely, higher average values were observed in the arm tissue for samples corresponding to medium- and large-sized specimens. In spite of such differences among the tissues considered, present ω3-PUFA levels in specimens of all size groups can be considered to be highly valuable regarding nutritional and healthy requirements [2,4,8].

Previous research has also shown high values of ω3-PUFAs in non-edible zones of the present species of octopus. Expressed as g·100 g^−1^ total FAs, the ovary and digestive gland depicted the 40.4–47.0 and 35.8–43.5 ranges, respectively, during a seasonal study of Atlantic octopus (*O. vulgaris*) [32]. A recent lipid extraction with low-toxicity solvents revealed values included in the 36.8–38.3 range for octopus (*O. vulgaris*) by-products [33]. Concerning non-edible tissues of other related cephalopod species, Kacem et al. [36] showed a 21.4–26.1 range in cuttlefish (*S. officinalis*) viscera from two different catching times and a ca. 37.4 value was reported for total viscera of squid (*I. argentinus*) [37]. A seasonal study on total squid (*D. gahi*) by-products indicated a 46.1–48.6 range [34].

Previous studies have also reported a high ω3-PUFA presence in edible tissues corresponding to the current species of octopus. Thus, similar values (expressed as g·100 g^−1^ total FAs) were detected in the edible tissue considered as a whole by Bonafe et al. [42] (39.4), Özoğul et al. [39] (41–47 range), and Zlatanos et al. [41] (37.7). Regarding single tissues, arms corresponding to octopus (*O. vulgaris*) captured in the Mediterranean Sea revealed a 46–49 range value [38]. The seasonal study of the ω3-PUFA presence showed ranges of 49.8–54.2 and 50.3–55.3 for arm and mantle, respectively, corresponding to specimens obtained in the European Atlantic coast [32]. Conversely, a low ω3-PUFA value (17.3) was detected by Biandolino et al. [45] in the edible tissue of farmed specimens from the Ionian Sea.

#### 2.1.2. Total ω3/Total ω6 FA Ratio

It is well-known that Western countries, in general, do not consume necessary levels of ω3-PUFAs, so a great attention has been accorded to the ω3/ω6 ratio of foods included in the human diet [3,4]. With the aim of preventing several health disorders (cardiovascular, neurological, and inflammatory), the World Health Organization (WHO) recommends nowadays a higher ratio than 1:10 in the human diet [46]. Furthermore, the European Nutritional Society indicated that notable health benefits could be achieved if an ω3/ω6 ratio of 1:5 or higher was provided in the human diet [47].

**Figure 2:**
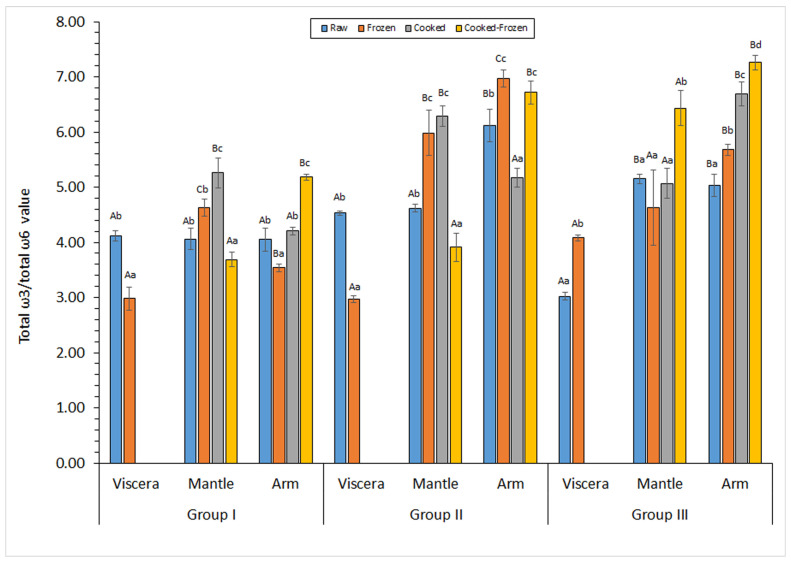
Determination of the total ω3 polyunsaturated fatty acid (ω3-PUFA) content (g·100 g^−1^ total FAs) in different kinds of raw and processed tissues corresponding to different specimen sizes. Mean values of three replicates (*n* = 3); standard deviations are indicated by bars. Within each group and for each raw/processed substrate, different capital letters (A,B,C) indicate significant differences (*p* < 0.05) among tissues. Within each group and for each tissue, different lowercase letters (a,b,c,d) indicate significant differences (*p* < 0.05) as a result of processing. Specimen groups are as expressed in Table 1. The viscera tissue was not subjected to the cooking process.

Values for ω3/ω6 ratios obtained in raw samples in the current study were included in the 3.0–4.5, 4.1–5.2, and 4.1–6.1 ranges for viscera, mantle, and arm tissues, respectively (Figure 2). The analysis of the ω3/ω6 ratio values revealed no differences (*p* > 0.05) among tissues in specimens corresponding to the small-sized group. Conversely, the arm tissue depicted higher levels (*p* < 0.05) than its counterpart viscera tissue for medium- and large-sized specimens. In the case of large-sized samples, the mantle tissue provided a higher ω3/ω6 ratio value (*p* < 0.05) than the viscera one. In spite of differences mentioned, and in agreement with current recommendations provided by nutritional organizations, ω3/ω6 ratio values obtained in all cases can be considered as highly valuable for the human diet.

Compared to the present values obtained for viscera, Sieiro et al. [32] observed higher ω3/ω6 ratio values for the ovary and the digestive gland (4.2–8.3 and 5.0–9.1 ranges, respectively) during a seasonal study of common octopus (*O. vulgaris*). Conversely, total by-products from the same marine species showed values included in the 3.2–3.7 range when extracted with low-toxicity solvents (ethanol, acetone, and methyl acetate) [33]. In the case of other cephalopod species, higher ω3/ω6 ratio values were detected for squid (*I. argentinus*) viscera (7.6–8.0 range) [37] and squid (*D. gahi*) by-products (12.1–13.3 range) [34] than in the present viscera tissue. Conversely, lower values (1.5–2.4 range) were detected by Kacem et al. [36] for cuttlefish (*S. officinalis*) viscera from specimens corresponding to two different catching times.

Higher ω3/ω6 ratios than found in the current work were obtained during a seasonal study of different edible tissues obtained from Atlantic European octopus (*O. vulgaris*) [32], in which arm and mantle tissues provided values included in the 9.5–12.8 range. Similar values to those in the present research were reported for the same species in the muscle tissue by Oliveira et al. [43] (5.3), Özoğul et al. [38] (3.8–5.6), and Zlatanos et al. [41] (3.6). Conversely, Biandolino et al. [45] showed a notably lower ω3/ω6 ratio value (1.7) in the arm tissue of farmed octopus (*O. vulgaris*).

#### 2.1.3. Single ω3-PUFAs

Among ω3-PUFAs, EPA and DHA have received a great attention, in agreement with their beneficial health properties. Thus, EPA consumption has been associated with low prevalence of circulatory, coronary, and inflammatory diseases [48]. On the other side, DHA has been reported to be responsible for the prevention of neurodegenerative diseases and correct fetal development, and functioning of the nervous system and visual organs in the fetus [49]. In spite of its lower content in seafood than EPA and DHA, DPA is attracting an increasing attention because of its presence in human brain and its high levels in human milk, which implies a potential impact during pregnancy and early development [50,51]. Moreover, DPA has been reported to be related to the improvement of cardiovascular and metabolic diseases [52,53]. Consequently, and on the basis of their great significance to human health, the current research on the FA profile will now be focused on the presence of these three ω3-PUFAs, i.e., EPA, DHA, and DPA.

In the present study, the EPA value was included in the 19.6–21.9, 18.4–18.8, and 17.2–19.3 g·100 g^−1^ total FAs ranges for viscera, mantle, and arm tissues, respectively, in the different size groups (Table 4). The following decreasing sequence (*p* < 0.05) was observed for the EPA content of tissues corresponding to small-sized specimens: viscera > mantle > arm. In the case of medium- and large-sized groups, the lowest values (*p* < 0.05) were detected in the mantle, while the highest average levels were obtained in the viscera tissue.

Results concerning DHA are depicted in Table 5. A notably lower value (*p* < 0.05) (expressed as g·100 g^−1^ total FAs) was detected for all sizes (Groups I, II, and III) in the viscera tissue (12.7–17.5 range) than in the edible tissues. Differences between arm (24.3–30.1 range) and mantle (24.3–26.1 range) values were only observed in medium-sized samples, with arm tissue showing a higher (*p* < 0.05) content.

Regarding DPA, values were notably lower than in the case of the two other ω3-PUFAs (Table 6). Thus, ranges of 2.6–3.5, 1.5–1.9, and 1.9–2.5 g·100 g^−1^ total FAs were obtained for viscera, mantle, and arm tissues, respectively, in the different size groups. The lowest average values were detected in the mantle tissue for all sizes of specimens; differences with other tissues were found significant (*p* < 0.05) for Groups II and III. The highest average values were obtained in viscera; remarkably, differences with the arm tissue were significant (*p* < 0.05) in small- and large-sized samples.

Previous research regarding non-edible tissues of the current species of octopus showed a different decreasing tendency (i.e., DHA > EPA > DPA) than in the current study for the presence of the three ω3-PUFAs. Thus, the following ranges were detected during a seasonal study [32]: 24.8–31.6, 13.4–18.1, and 0.3–1.1 g·100 g^−1^ total FAs, respectively, in ovary tissue; in the same study [32], the digestive gland provided the following value ranges: 16.7–28.7, 14.4–18.1, and 0.1–1.0 g·100 g^−1^ total FAs, respectively. In agreement with the present research, the EPA value (21.0–22.4 g·100 g^−1^ total FAs range) showed to be higher than that of DHA (14.0–14.5 g·100 g^−1^ total FAs range) in lipid extracts obtained from total by-products by employing low-toxicity solvents (ethanol, acetone, and ethyl acetate) [33]. In the case of other cephalopod species, total viscera obtained from squid (*I. argentinus*) revealed values ca. 16.4, 9.3, and 0.5 g·100 g^−1^ total FAs for DHA, EPA, and DPA, respectively [37]. During a seasonal study carried out on Patagonian squid (*D. gahi*) by-products [34], 29.5–30.8, 15.9–17.2, and 0.5–0.6 g·100 g^−1^ total FAs ranges were detected for DHA, EPA, and DPA, respectively. A similar distribution (35.0–39.0, 13.1–14.3, and 0.7–0.8 g·100 g^−1^ total FAs ranges, respectively) was observed in the arms and tentacles of European squid (*Loligo vulgaris*) [54]. Conversely, a seasonal study carried out on cuttlefish (*S. officinalis*) viscera led to ranges of 6.3–9.1, 7.1–11.6, and 1.66 g·100 g^−1^ total FAs for DHA, EPA and DPA.

Previous research has already shown the current decreasing tendency, i.e., DHA > EPA > DPA, in the edible tissues of the present species of octopus. Thus, Zlatanos et al. [41] detected values of 20.1, 13.6, and 2.0 g·100 g^−1^ total FAs, respectively, in muscle tissue. In a seasonal study [32], ranges of 28.3–32.9, 19.1–21.4, and 0.2–0.5 g·100 g^−1^ total FAs, respectively, were detected in arm and mantle tissues. Oliveira et al. [43] showed values of 100.4, 77.2, and 7.0 mg·100 g^−1^ edible tissue, respectively. In a different species of octopus, *Eledone moschata*, values of 24.7, 16.7, and 1.8 g·100 g^−1^ total FAs, respectively, were detected in the mantle tissue [55]. During a seasonal study of musky octopus (*E. moschata*) [39], the edible tissue showed values included in the 21.0–28.2 and 7.9–12.2 g·100 g^−1^ total FAs ranges for DHA and EPA, respectively.

### 2.2. Effect of Cooking on the FA Composition

The cooking treatment was applied to raw mantle and arm tissues but not to viscera. As for samples corresponding to the raw stage (Section 3.1), changes produced in the FA profile will be discussed on the basis of the cooking effect on FA groups and ratios as well as on single ω3-PUFAs.

#### 2.2.1. FA Groups

As a result of the thermal treatment, an increased average value of the STFA presence was observed in all tissues and for specimens corresponding to all sizes (Table 1). Differences were found to be significant (*p* < 0.05) in the mantle of medium-sized samples and in the arm of small-sized samples. In the case of MUFAs (Table 2), the evaluation of the average values did not provide any definite tendency and no effect (*p* > 0.05) of the cooking process could be implied on any of the tissues for any of the sizes considered. A general decrease in the average PUFA level was found after the cooking process (Table 3). This decrease was found to be significant (*p* < 0.05) in the mantle of medium-sized specimens and in the arm of small-sized specimens. Regarding the ω3-PUFA value (Figure 1), the only significant change (*p* < 0.05) detected was a content increase in the mantle tissue of medium-sized samples.

#### 2.2.2. FA Ratios

The ω3/ω6 ratio showed an average increase with the cooking process in small-sized specimens, with differences being significant (*p* < 0.05) in the mantle tissue (Figure 2). A definite trend could not be concluded in the two other tissues, although increases in this ratio were obtained in the mantle of medium-sized specimens and in the arm of large-sized samples.

The PUFA presence in the human diet has been found to be strongly related to nutritional value, digestibility, and preserving properties [56,57]. In order to evaluate the PUFA content variation during seafood processing and storage, the polyene index (PI), defined as the DHA+EPA/C16:0 ratio of FA concentrations, has widely been employed [37,58]. Technologists focused on seafood consider this ratio to be a valuable and practical tool for the assessment of the lipid fraction damage and therefore, to the quality loss of seafood during different steps of processing.

**Figure 3:**
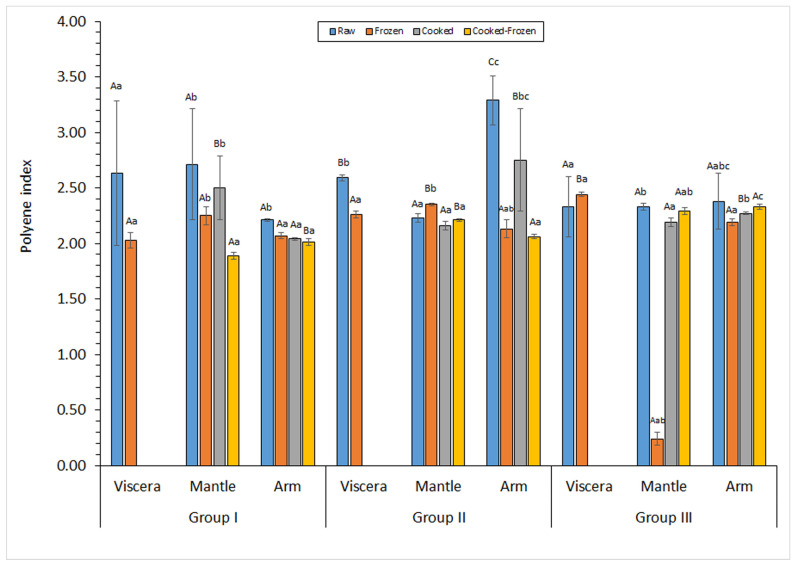
Determination of the total ω3 polyunsaturated fatty acid (ω3-PUFA) content (g·100 g^−1^ total FAs) in different kinds of raw and processed tissues corresponding to different specimen sizes. Mean values of three replicates (*n* = 3); standard deviations are indicated by bars. Within each group and for each raw/processed substrate, different capital letters (A,B,C) indicate significant differences (*p* < 0.05) among tissues. Within each group and for each tissue, different lowercase letters (a,b,c) indicate significant differences (*p* < 0.05) as a result of processing. Specimen groups are as expressed in Table 1. The viscera tissue was not subjected to the cooking process.

PI values obtained in the present research are expressed in Figure 3. Raw samples showed values included in the 2.3–2.6, 2.2–2.7, and 2.2–3.3 ranges for viscera, mantle and arm tissues, respectively. No differences (*p* > 0.05) among tissues could be detected in small- and large-sized specimens (Figure 3). Conversely, the following decreasing sequence was observed in medium-sized specimens: arm > viscera > mantle. Regarding the effect of the cooking process, the average PI revealed a general decrease (Figure 3). This decrease was significant (*p* < 0.05) in the arms of small-sized samples and in the large-sized samples from the mantle tissue.

The FLQ index has also been employed in order to assess the quality of the dietary lipid source [59,60,61]. FLQ values obtained in the current study are presented in Table 7. Raw samples depicted values included in the 47.7–65.0, 74.4–80.9 and 73.5–99.2 ranges for viscera, mantle, and arm tissues, respectively. In all sizes of specimens, a lower value (*p* < 0.05) was depicted in viscera than in both edible tissues. The cooking process did not lead to a general trend in the mantle tissues. Thus, decreased values (*p* < 0.05) were detected in frozen small- and medium-sized samples and an increase (*p* < 0.05) in frozen big-sized specimens was obtained. Regarding the arm tissue, an increase (*p* < 0.05) in frozen small-sized samples and a decrease (*p* < 0.05) in raw medium-sized specimens were observed.

Current values obtained for mantle and arm tissues have shown to be higher than those reported for muscle corresponding to freshwater and farmed fish species. Thus, FLQ value of ca. 34.8, 37.2, and 41.3 were detected for roach (*Rutilus rutilus*), perch (*Perca fluviatilis*), and pike (*Esox lucius*) muscle [61]. During a seasonal study, Senso et al. [59] reported a 19.4–31.3 value range for farmed gilthead sea bream (*Sparus aurata*). A ca. 24.5–36.4 value range was obtained in several freshwater fish species (roach, *R. rutilus*; bream, *Abramis brama*; pike, *E. lucius*; Eurasian perch, *P. fluviatilis*) [60].

#### 2.2.3. Single ω3-PUFAs

Increased EPA values (*p* < 0.05) were obtained with the cooking process for the arm of small-sized octopus and for the mantle of medium-sized samples (Table 4); conversely, large-sized specimens revealed a decrease (*p* < 0.05) in the mantle tissue. Concerning the DHA value (Table 5), a general increase in the average value with cooking was obtained in the mantle of specimens corresponding to all sizes; this increase was found to be significant (*p* < 0.05) in the mantle of large-sized samples. For the arm tissue, a definite trend could not be concluded for the DHA level. Regarding the DPA presence, a decreased value (*p* < 0.05) was obtained in the mantle tissue of small- and large-sized samples and in the arm tissue of large-sized samples; conversely, small-sized specimens showed a DPA level decrease in the arm tissue.

#### 2.2.4. Previous Related Studies Regarding the Effect of Cooking on Cephalopod Tissues

Different damage pathways have been pointed out in previous studies, resulting from the cooking treatment of seafood in general. Among them, heat degradation of nutrients, oxidation of vitamins and lipids, and protein toughening can be mentioned [62,63]. As a result, nutritional and sensory quality losses have been indicated, especially when over-processing is carried out. In such studies, great attention has been given to the evolution of PUFA compounds, as being especially prone to lipid oxidation development [58] and consequently, lead to a wide range of negative effects on nutritional and healthy values [64,65].

Previous work related to the effect of cooking on non-edible tissues corresponding to cephalopod species can be considered scarce. Toyes-Vargas et al. [44] detected the loss of FA values after the cooking process of Giant squid (*D. gigas*) viscera; thus, decreased values (g·kg^−1^ dry tissue weight) were detected for EPA (from 16.9 to 14.0), DHA (from 19.4 to 18.9), C20:4ω6 (from 3.6 to 3.4), and total STFAs (from 38.2 to 31.2). A decreasing tendency for the DHA, EPA and PI values was detected in squid (*I. argentinus*) viscera when increasing the heating time and the temperature of processing [37]; conversely, the authors observed no effect on the ω3/ω6 ratio.

Previous research regarding the effect of the cooking process and thermal treatment, in general, on the FA profile of edible tissues of cephalopod species can also be considered scarce. A marked effect on the FA profile of octopus (*O. vulgaris*) muscle was observed by Czech et al. [66] after subjecting it to a frying process with sunflower oil. The authors observed a remarkable content decrease (g·100 g^−1^ total FAs) in the fried product in EPA (from 14.0 to 0.5), DHA (from 29.6 to 2.5) and DPA (from 0.9 to 0.1) values and in the ω3/ω6 ratio (from 4.0 to 0.1). No differential losses in FA groups were detected by Oliveira et al. [43] by subjecting gutted octopus (*O. vulgaris*) to the boiling process. Thus, losses of 89.5%, 92.1%, 89.0% and 88.9% for STFA, MUFA, PUFA, and ω3-PUFA groups, respectively, were detected; additionally, a decrease from 5.25 to 5.17 was produced for the ω3/ω6 ratio and from 2.44 to 2.34 for the PI as a result of the thermal treatment.

### 2.3. Effect of Frozen Storage on the FA Composition

The effect of the frozen storage was studied on the raw samples of the viscera, mantle, and arm tissues and on the cooked samples of the mantle and arm tissues. As for samples corresponding to the raw and cooked conditions, changes produced in the FA profile will be discussed on the basis of the effect of the frozen storage on FA groups and ratios, as well as on single ω3-PUFAs.

#### 2.3.1. FA Groups

Frozen storage led to a significant increase in STFA content in viscera (Table 1); this difference was significant (*p* < 0.05) in specimens corresponding to the large-sized group. Regarding mantle and arm tissues, a notable increase in the average STFA content was detected in all size groups as a result of the frozen storage. This tendency was observed both for raw as for cooked samples.

The effect of frozen storage on MUFA content varied by sample size: it increased significantly (p < 0.05) in small-sized viscera but decreased (*p* < 0.05) in large-sized ones. Meantime, the mantle tissue showed no effect (*p* > 0.05) for small- and medium-sized specimens; however, an average decrease was found in large-sized specimens, which was significant (*p* < 0.05) for cooked samples. A different behavior was obtained for the arm tissue according to the size of specimens. Thus, small- and large-sized samples showed a decrease in the average value, while medium-sized samples experienced an increase; a significant effect (*p* < 0.05) of the frozen period was detected in cooked small- and large-sized groups.

A decrease (*p* < 0.05) of the PUFA value was detected in viscera corresponding to the small- and medium-sized samples as a result of frozen storage; conversely, an average value increase was observed for large-sized specimens. Regarding the mantle tissue, a general decrease in the average PUFA level was observed; this decrease was found to be significant (*p* < 0.05) in medium-sized samples both for raw and cooked samples. A general decrease in the average PUFA value was observed in the arm tissue; this decrease was significant (*p* < 0.05) for small- and medium-sized specimens corresponding to the cooked condition.

A decrease (*p* < 0.05) in the total ω3-PUFA value was observed in viscera from small- and medium-sized samples; conversely, large-sized samples saw an increase (*p* < 0.05) as a result of the frozen period. Mantel and arm tissues showed a decrease (*p* < 0.05) of the ω3-PUFA value in raw and cooked samples of Group II. However, a definite trend for both tissues could not be concluded for this FA group regarding specimens corresponding to Groups I and III.

#### 2.3.2. FA Ratios

A notable decrease (*p* < 0.05) in the ω3/ω6 ratio was observed as a result of the storage period in the viscera tissue corresponding to small- and medium-sized samples. Conversely, viscera corresponding to large-sized octopus showed an increased value (*p* < 0.05). Regarding the mantle tissue, a decreased value (*p* < 0.05) was obtained for cooked small- and medium-sized samples, but an increased value (*p* < 0.05) was detected in raw medium-sized samples and cooked large-sized samples. For the arm tissue, an increased value (*p* < 0.05) was obtained in most cases; the exception was the raw small-sized batch that exhibited a decrease (*p* < 0.05) after the storage period.

The PI of the viscera tissue indicated an average decrease for specimens corresponding to the small- and medium-sized batches; differences were significant (*p* < 0.05) for the medium-sized ones. Small-sized specimens showed an average PI value decrease in the mantle and arm tissues as a result of the frozen storage; differences were significant (*p* < 0.05) for cooked mantle and raw arm. Concerning medium- and large-sized samples, an increased PI was observed after the frozen period in the mantle tissue in most cases; differences were significant (*p* < 0.05) for raw medium-sized samples. For the arm tissue, a general decrease in the average PI was detected in medium- and large-sized samples; conversely, cooked specimens corresponding to Group III showed a content increase (*p* < 0.05) with frozen storage.

The FLQ value of the viscera tissue indicated a significant decrease (*p* < 0.05) in specimens of all sizes (Table 7). The effect of the frozen storage on the mantle tissue did not lead to a general trend. Thus, a value decrease (*p* < 0.05) was detected in cooked small- and medium-sized samples and a value increase in raw medium-sized specimens. For the arm tissue, decreased values were obtained in raw small- and medium-sized specimens.

#### 2.3.3. Single ω3-PUFAs

Regarding the EPA value, viscera samples showed an average decrease with the frozen storage; differences were significant (*p* < 0.05) in small- and medium-sized samples. For mantle samples, a decreased value was detected after the frozen storage in most cases; differences were significant (*p* < 0.05) in raw and cooked small-sized samples and in cooked medium-sized ones. Conversely, the mantle tissue corresponding to raw medium-sized and cooked large-sized samples showed an increased (*p* < 0.05) EPA level as a result of the frozen storage. In the case of the arm tissue, the effect of frozen storage varied by specimen size. Thus, small- and large-sized samples depicted an average decrease, while medium-sized ones indicated an increase.

The DHA value in viscera showed an increase (*p* < 0.05) after the frozen storage in medium- and large-sized samples. Regarding the mantle tissue, an average value decrease was detected for this PUFA group in most cases; differences were found to be significant (*p* < 0.05) in cooked small- and medium-sized batches. A decreased DHA value was generally observed in the arm tissue of small- and medium-sized samples after the frozen period; this decrease was found to be significant (*p* < 0.05) in raw samples. Regarding large-sized octopus, an increasing average DHA content was observed in the arm tissue as a result of the frozen storage; differences were significant (*p* < 0.05) in cooked samples.

For the DPA level, a different trend was detected according to the size of specimens; that is, the viscera tissue showed a decrease (*p* < 0.05) in small- and medium-sized samples and an increase (*p* < 0.05) in large-sized ones. Regarding the mantle tissue, an average increase was seen in most cases. Conversely, an average decrease in the DPA value was detected in the arm tissue of all groups as a result of frozen storage; differences were only significant (*p* < 0.05) in cooked medium-sized samples.

#### 2.3.4. Previous Related Studies Regarding the Effect of Frozen Storage on Cephalopod and Invertebrate Tissues

Frozen storage mostly inhibits microbial development, but fish constituents may undergo different kinds of deteriorative mechanisms such as the formation of aggregates, protein insolubility, mechanical damage, and development of lipid damage [67,68]. Among them, lipid hydrolysis and oxidation, and therefore FA damage, have been recognized as important factors that influence quality changes during the frozen storage of seafood in general [69,70]. Notably, the development of the lipid damage mechanisms has been revealed to be more important when increasing the time and temperature of the storage period [15,71].

Regarding non-edible tissues of cephalopods and invertebrate species in general, previous research focused on the effect of the frozen storage on changes in the FA profile, and lipid damage was considered scarce. A marked development of lipid oxidation was detected in Patagonian squid (*D. gahi*) by-products during the frozen storage (−10 and −18 °C up to 18 months) [72]; in this study, lipid deterioration increased with the time and temperature of storage. Regarding invertebrate species, the evolution of the FA profile of Chinese mitten crab (*Eriocheir sinensis*) hepatopancreas during the frozen storage at different temperatures (−20, −40, and −80 °C) was studied by Fan et al. [73]; an increase in the STFA level and a decrease in the MUFA and PUFA presence were detected with increasing temperature and time of storage.

Previous research accounts for the study of changes in the FA profile in different edible tissues of cephalopod species subjected to frozen storage conditions. Thus, Atayeter and Ercoşkun [54] analyzed the changes produced in the FA profile of the arms and mantle of European squid (*L. vulgaris*) subjected to frozen storage (−20, −40, and −80 °C). By increasing the storage time, a marked increase in the STFA presence could be detected, which was accompanied by slight decreases in the MUFA and PUFA values and remarkable increases in the ω3/ω6 ratio; additionally, marked decreases in the DHA and DPA levels were detected, but no effect on the EPA value could be proved. Gullian-Klan et al. [74] analyzed the changes produced in the FA profile of Mexican four-eyed octopus (*Octopus maya*) muscle during a 5-month storage at −18 °C. As a result, 6.07% and 9.28% decreases in the PUFA value were detected after 3 and 5 months of storage, respectively. Furthermore, the initial PI value (i.e., 3.5) decreased to values of 2.9 and 2.3 after 3 and 5 months, respectively.

## 3. Materials and Methods

### 3.1. Raw Octopus, Sampling, Cooking and Frozen Storage

Common octopus (*O. vulgaris*) were obtained near the Galician coast (North-West Spain) and supplied by Frigoríficos Rosa de los Vientos S. L. (Marín, Pontevedra, Spain). Three different specimen groups were considered, i.e., Group I (1–2 kg per specimen), Group II (2–3 kg per specimen), and Group III (3–4 kg per specimen). In order to carry out the study, 24 specimens of each group were used. Within each group, separation of viscera, mantle and arm was carried out.

Viscera tissue corresponding to 12 specimens was subjected to analysis (Raw viscera). For this, three independent batches were considered (*n* = 3; viscera corresponding to 4 specimens per batch). Viscera tissue corresponding to the other 12 specimens was subjected to frozen storage (−18 °C) for 4 months. After this period, viscera tissue was subjected to thawing and subsequent analysis (Frozen viscera). For this, three independent batches were considered (*n* = 3; viscera corresponding to 4 specimens per batch). In agreement with industrial practice, the viscera tissue was not subjected to the cooking process.

Mantle tissue corresponding to 6 specimens was subjected to analysis (Raw mantle). For this, three independent batches were considered (*n* = 3; mantles corresponding to 2 specimens per batch). Meantime, mantle tissue corresponding to 6 specimens was subjected to frozen storage (−18 °C) for 4 months. After this period, mantle tissue was subjected to thawing and subsequent analysis (Frozen mantle). For this, three independent batches were considered (*n* = 3; mantles corresponding to 2 specimens per batch).

Mantle tissue corresponding to the remaining 12 specimens was subjected to cooking (40 min at 90 °C). Afterwards, mantle tissue corresponding to 6 specimens was subjected to analysis (Cooked mantle). For this, three independent batches were taken into account (*n* = 3; mantles corresponding to 2 specimens per batch). Meantime, mantle tissue corresponding to 6 specimens was subjected to frozen storage (–18 °C) for 4 months. After this period, the mantle tissue was subjected to thawing and subsequent analysis (Cooked-Frozen mantle). For this, three independent batches were considered (*n* = 3; mantles corresponding to 2 specimens per batch).

For the arm tissue, the sampling procedure carried out was the same as for the mantle tissue. Thus, the following arm samples were obtained: Raw, Frozen, Cooked and Cooked-Frozen. As for the mantle tissue, three independent batches were considered (*n* = 3; arms corresponding to 2 specimens per batch) in all sample types.

Solvents and chemical reagents used in this study were of reagent grade (Merck, Darmstadt, Germany); otherwise, the supplier is defined.

### 3.2. Lipid Extraction and FA Analysis of Tissue Samples

The lipid extraction of the different tissues was carried out in agreement with the Bligh and Dyer [75] method. In a first step, this method employs a chloroform/methanol/water (1/2/0.8, *v/v/v*) mixture as an extracting solvent system. Then, the addition of chloroform and water is carried out to the mixture in order to attain a 2/2/1.8 (chloroform/methanol/water, *v/v/v*) solvent ratio so that two phases are formed. The one placed at the bottom is carefully taken and corresponds to the lipid fraction. Results on total lipid yield were calculated as g total lipids·kg^−1^ tissue. Lipid extracts were kept at −40 °C in a nitrogen atmosphere before being used.

Fatty acid methyl esters (FAMEs) were obtained from lipid extracts by employing acetyl chloride in methanol. Then, FAMEs were analyzed by gas chromatography (Perkin-Elmer 8700 chromatograph, Madrid, Spain) [76]. A fused silica capillary column SP-2330 (0.25 mm i.d. × 30 m, Supelco, Inc., Bellefonte, PA, USA) was used. The temperature program was the following: increased from 145 to 190 °C at 1.0 °C·min^−1^ and from 190 °C to 210 °C at 5.0 °C·min^−1^, then held for 13.5 min at 210 °C. Nitrogen at 10 psig was used as carried gas and a flame ionization detector at 250 °C was used as detector. A programmed temperature vaporizer injector was used in the split mode (150:1), being heated from 45 to 275 °C at 15 °C·min^−1^.

Identification of FAME peaks was carried out by comparison of the retention times to those of standard mixtures (Qualmix Fish, Larodan, Malmo, Sweden; Supelco 37 Component FAME Mix, Sigma-Aldrich, Laramie, WY, USA). Peak areas were automatically integrated. For quantitative purposes, C19:0 was employed as an internal standard; for that, 100 μL (i.e., 40 μg C19:0) of a 0.4 mg·mL^−1^ solution in toluene were added to each sample before the methylation reaction with acetyl chloride in methanol. Detection and quantification limits were 500 and 1,500 area units, respectively. Quantitative calibration was carried out by means of the above-mentioned Supelco FAME Mix.

The content of each FA was expressed as g·100 g^−1^ of total FAs. Results regarding FA groups (STFAs, MUFAs, PUFAs, total ω3-PUFAs and total ω6-PUFAs) and FA ratios (total ω3-PUFAs/total ω6-PUFAs, PI, and FLQ) were calculated by considering the results obtained in individual FAs. The PI and the FLQ were determined as the following ratios of FA concentrations: DHA+EPA/C16:0 and 100 × (DHA + EPA)/% total FAs, respectively.

### 3.3. Statistical Analysis

This research was carried out in triplicate (*n* = 3). For each kind of sample (raw/cooked/frozen, specimen size, and tissue), three biological replicates (three independent batches) were employed. In the case of mantle and arm, each sample analyzed was composed of tissues corresponding to two different specimens. For viscera, each sample analyzed was composed of tissues corresponding to four different specimens.

Data obtained from the FA analysis (FA groups and ratios and single ω3-PUFAs) were subjected to the ANOVA method. For it, one-way ANOVA was applied to investigate differences resulting from the following factors: tissue, cooking process and frozen storage. The effect of each factor was analyzed independently. Statistical comparisons were conducted via PASW Statistics 18 Software for Windows (SPSS Inc., Chicago, IL, USA). The least-squares difference (LSD) test was used for comparison of means. For each FA parameter, the 95% confidence interval was calculated; for this, the standard deviation of each sample and the number of replicates (*n* = 3) were taken into account.

## 4. Conclusions

A comparative study of the FA composition of non-edible (viscera) and edible (mantle and arm) tissues of octopus (*O. vulgaris*) was carried out. In agreement with current nutritional recommendations, all kinds of tissues (viscera, mantle, and arm) showed great levels of highly valuable indices related to the lipid fraction (total ω3-PUFAs, EPA, DHA, and DPA values; ω3/ω6 PI, and FLQ ratios). In most cases, the cooking process and the frozen storage led to an average decrease in the PUFA and ω3-PUFA content and to an increase in the STFA presence. In spite of such processing effects, all kinds of specimen sizes considered in the present study maintained such highly nutritional values. This result is considered especially important in the case of the viscera tissue, a substrate that is commonly discarded or employed for obtaining low-value sub-products.

This research contributes to achieve alternative sources for obtaining highly valuable constituents from waste substrates resulting from the seafood commercialization with the aim of providing healthy compounds for the food and pharmaceutical industries and increase the profitability of such by-products. Waste constituents may be used not only as food but also for other high-end applications. The results of this study could serve as a basis for the development of new functional foods enriched with ω3-PUFAs such as EPA and DHA. Such enriched foods would be likely to produce a positive and profitable impact on the health of a wide range of consumers such as infants, pregnant women, or older adults in general.

The use of the current viscera tissue would lead to the ecological upside of reducing seafood industry waste and agree with general commitments for environmental sustainability and circular economy. Further research ought to be carried out on the optimization (i.e., surface-response methodology) of the extracting conditions and considering the different process and response variables of the extraction. The use of an RSM design can provide the possibility of affording a product with, at the same time, a minimum rancidity level and maximum yield of ω3-PUFA compounds. For it, the employment of green technologies such as irradiation (microwave, ultrasound, pulsed electric fields, etc.)-assisted, supercritical fluid, or green solvent extraction ought to be developed to fulfil current ecological requirements and guarantee a high-quality ω3-PUFA extract. Before finding a subsequent use for octopus waste, international requirements regarding safety concerns (presence of heavy metals, aromatic hydrocarbons, etc.) ought to be taken into account.

## Figures and Tables

**Table 1:** Saturated fatty acid (STFA) content (g·100 g^−1^ total FAs) * in different kinds of raw and processed tissues corresponding to different specimen sizes **.

Specimen Size	Raw or Processed Tissue	Tissue		
		Viscera	Mantle	Arm
Group I	Raw	31.82 ± 3.33 Aa	31.93 ± 2.82 Aa	34.48 ± 0.09 Aa
	Frozen	32.56 ± 0.41 Aa	36.27 ± 0.61 Bb	35.92 ± 0.11 Bc
	Cooked	NA ***	33.74 ± 1.81 Aab	35.05 ± 0.34 Ab
	Cooked-Frozen	NA	37.70 ± 0.35 Ac	37.54 ± 0.18 Ad
Group II	Raw	31.83 ± 0.23 Ba	35.14 ± 0.43 Ca	28.82 ± 0.94 Aa
	Frozen	32.47 ± 0.35 Aa	36.63 ± 0.12 Bb	37.90 ± 0.60 Cb
	Cooked	NA	36.49 ± 0.38 Bb	31.30 ± 2.84 Aa
	Cooked-Frozen	NA	37.06 ± 0.05 Ac	37.94 ± 0.13 Ab
Group III	Raw	29.16 ± 1.49 Aa	35.51 ± 0.24 Ba	34.71 ± 1.15 Ba
	Frozen	32.42 ± 0.19 Ab	36.17 ± 0.16 Bb	36.80 ± 0.02 Bb
	Cooked	NA	35.72 ± 0.37 Aab	35.79 ± 0.14 Aa
	Cooked-Frozen	NA	37.53 ± 0.29 Ac	37.06 ± 0.18 Ab

* Average values± standard deviations of three replicates (*n* = 3). Within each group and for each row, different capital letters (A–C) indicate significant differences (*p* < 0.05) among tissues. Within each group and for each column, different lowercase letters (a–d) indicate significant differences (*p* < 0.05) as a result of processing. ** Specimen sizes: Group I (1–2 kg per specimen), Group II (2–3 kg per specimen), and Group III (3–4 kg per specimen). *** NA: not analyzed.

**Table 2:** Monounsaturated fatty acid (MUFA) content (g·100 g^−1^ total FAs) * in different kinds of raw and processed tissues corresponding to different specimen sizes **.

Specimen Size	Raw or Processed Tissue	Tissue		
		Viscera	Mantle	Arm
Group I	Raw	18.01 ± 0.92 Ba	10.05 ± 0.08 Aa	10.29 ± 0.03 Ab
	Frozen	20.40 ± 0.88 Bb	10.14 ± 0.04 Aa	10.26 ± 0.28 Ab
	Cooked	NA ***	10.17 ± 0.33 Aa	10.24 ± 0.44 Ab
	Cooked-Frozen	NA	10.32 ± 0.10 Ba	9.14 ± 0.17 Aa
Group II	Raw	17.03 ± 0.04 Ba	10.90 ± 0.27 Aa	10.0 ± 0.46 Aa
	Frozen	17.03 ± 44 Ba	10.15 ± 0.20 Aa	10.51 ± 0.29 Aa
	Cooked	NA	10.86 ± 0.33 Ba	9.97 ± 0.29 Aa
	Cooked-Frozen	NA	10.64 ± 0.08 Ba	10.02 ± 0.08 Aa
Group III	Raw	23.71 ± 0.65 Bb	10.43 ± 0.25 Ab	9.99 ± 0.31 Ab
	Frozen	18.42 ± 0.25 Ba	10.11 ± 0.07 Ab	9.82 ± 0.09 Ab
	Cooked	NA	10.30 ± 0.07 Ab	10.03 ± 0.14 Ab
	Cooked-Frozen	NA	9.22 ± 0.07 Ba	8.45 ± 0.13 Aa

* Average values± standard deviations of three replicates (*n* = 3). Within each group and for each row, different capital letters (A,B) indicate significant differences (*p* < 0.05) among tissues. Within each group and for each column, different lowercase letters (a,b) indicate significant differences (*p* < 0.05) as a result of processing. ** Specimen sizes as expressed in Table 1. *** NA: not analyzed.

**Table 3:** Polyunsaturated fatty acid (PUFA) content (g·100 g^−1^ total FAs) * in different kinds of raw and processed tissues corresponding to different specimen sizes **.

Specimen Size	Raw or Processed Tissue	Tissue		
		Viscera	Mantle	Arm
Group I	Raw	50.17 ± 2.24 Ab	58.03 ± 1.75 Cc	55.26 ± 0.12 Bc
	Frozen	47.04 ± 0.87 Aa	53.59 ± 0.64 Bb	53.79 ± 0.34 Ba
	Cooked	NA ***	56.10 ± 1.90 Abc	54.72 ± 0.17 Ab
	Cooked-Frozen	NA	51.98 ± 0.42 Aa	53.33 ± 0.31 Ba
Group II	Raw	51.14 ± 0.19 Ab	53.96 ± 0.20 Bc	61.10 ± 1.22 Cb
	Frozen	50.51 ± 0.13 Aa	53.22 ± 0.22 Bbc	51.59 ± 0.88 Aa
	Cooked	NA	52.65 ± 0.28 Aab	58.73 ± 3.09 Bb
	Cooked-Frozen	NA	52.30 ± 0.05 Ba	52.05 ± 0.04 Aa
Group III	Raw	47.12 ± 2.14 Aa	54.06 ± 0.46 Ba	55.30 ± 1.86 Bab
	Frozen	49.15 ± 0.18 Aa	53.72 ± 0.12 Ba	53.38 ± 0.11 Ba
	Cooked	NA	53.98 ± 0.32 Aa	54.48 ± 0.24 Ab
	Cooked-Frozen	NA	53.25 ± 0.34 Aa	54.18 ± 0.08 Bb

* Average values± standard deviations of three replicates (*n* = 3). Within each group and for each row, different capital letters (A,B,C) indicate significant differences (*p* < 0.05) among tissues. Within each group and for each column, different lowercase letters (a,b,c) indicate significant differences (*p* < 0.05) as a result of processing. ** Specimen sizes as expressed in Table 1. *** NA: not analyzed.

**Table 4:** Determination of the eicosapentaenoic acid (EPA) content (g·100 g^−1^ total FAs) * in different kinds of raw and processed tissues corresponding to different specimen sizes **.

Specimen Size	Raw or Processed Tissue	Tissue		
		Viscera	Mantle	Arm
Group I	Raw	20.83 ± 0.83 Cb	18.57 ± 0.24 Bc	17.22 ± 0.24 Ab
	Frozen	17.19 ± 1.00 Ba	17.33 ± 0.19 Bb	16.30 ± 0.25 Aa
	Cooked	NA ***	18.18 ± 0.25 Ac	18.49 ± 0.15 Ac
	Cooked-Frozen	NA	16.03 ± 0.59 Aa	18.29 ± 0.72 Bbc
Group II	Raw	21.93 ± 0.03 Cb	18.41 ± 0.25 Ab	19.73 ± 0.79 Bab
	Frozen	17.47 ± 0.19 Aa	20.20 ± 0.56 Bc	21.56 ± 1.13 Bb
	Cooked	NA	19.31 ± 0.36 Ac	19.06 ± 0.51 Aa
	Cooked-Frozen	NA	17.06 ± 0.76 Aa	19.39 ± 0.70 Ba
Group III	Raw	19.58 ± 0.62 Ba	18.75 ± 0.18 Abc	19.33 ± 0.14 Ba
	Frozen	19.15 ± 0.10 Ba	17.52 ± 1.03 Aab	19.19 ± 0.23 Ba
	Cooked	NA	17.91 ± 0.38 Aa	20.21 ± 0.17 Bb
	Cooked-Frozen	NA	19.10 ± 0.32 Ac	20.01 ± 0.03 Bb

* Average values± standard deviations of three replicates (*n* = 3). Within each Group and for each row, different capital letters (A,B,C) indicate significant differences (*p* < 0.05) among tissues. Within each Group and for each column, different lowercase letters (a,b,c) indicate significant differences (*p* < 0.05) as a result of processing. ** Specimen sizes as expressed in Table 1. *** NA: not analyzed.

**Table 5:** Determination of the docosahexaenoic acid (DHA) content (g·100 g^−1^ total FAs) * in different kinds of raw and processed tissues corresponding to different specimen sizes **.

Specimen Size	Raw or Processed Tissue	Tissue		
		Viscera	Mantle	Arm
Group I	Raw	16.01 ± 2.22 Aa	26.14 ± 2.21 Bbc	25.15 ± 0.15 Ba
	Frozen	15.53 ± 0.34 Aa	25.13 ± 0.63 Cb	23.77 ± 0.22 Bb
	Cooked	NA ***	27.29 ± 1.16 Bc	23.49 ± 1.08 Aab
	Cooked-Frozen	NA	23.43 ± 0.67 Aa	24.94 ± 1.12 Aab
Group II	Raw	17.46 ± 0.14 Aa	24.25 ± 0.35 Bb	30.06 ± 1.28 Cc
	Frozen	18.05 ± 0.09 Ab	24.46 ± 0.41 Bb	23.60 ± 1.38 Ba
	Cooked	NA	24.57 ± 0.15 Ab	27.44 ± 2.12 Abc
	Cooked-Frozen	NA	22.99 ± 0.57 Aa	25.69 ± 0.94 Bab
Group III	Raw	12.70 ± 1.05 Aa	24.50 ± 0.23 Ba	24.29 ± 1.34 Bab
	Frozen	16.8 ± 0.125 Ab	24.40 ± 1.03 Bab	25.74 ± 0.37 Bb
	Cooked	NA	25.66 ± 0.30 Ab	24.94 ± 0.15 Aa
	Cooked-Frozen	NA	25.20 ± 0.24 Ab	25.67 ± 0.08 Ab

* Average values ± standard deviations of three replicates (*n* = 3). Within each group and for each row, different capital letters (A,B,C) indicate significant differences (*p* < 0.05) among tissues. Within each group and for each column, different lowercase letters (a,b,c) indicate significant differences (*p* < 0.05) as a result of processing. ** Specimen sizes are as expressed in Table 1. *** NA: not analyzed.

**Table 6:** Determination of the docosapentaenoic acid (DPA) content (g·100 g^−1^ total FAs) * in different kinds of raw and processed tissues corresponding to different specimen sizes **.

Specimen Size	Raw or Processed Tissue	Tissue		
		Viscera	Mantle	Arm
Group I	Raw	3.54 ± 0.48 Bb	1.85 ± 0.11 Ab	1.93 ± 0.03 Aa
	Frozen	2.45 ± 0.10 Ca	1.61 ± 0.06 Aa	1.86 ± 0.05 Ba
	Cooked	NA ***	1.64 ± 0.01 Aa	2.26 ± 0.11 Bb
	Cooked-Frozen	NA	1.94 ± 0.16 Ab	2.21 ± 0.11 Ab
Group II	Raw	2.59 ± 0.05 Bb	1.54 ± 0.03 Aa	2.50 ± 0.45 Bab
	Frozen	2.25 ± 0.05 Ba	1.99 ± 0.02 Ab	1.91 ± 0.09 Aa
	Cooked	NA	1.59 ± 0.15 Aa	2.76 ± 0.20 Bb
	Cooked-Frozen	NA	2.02 ± 0.02 Ab	1.95 ± 0.07 Aa
Group III	Raw	3.12 ± 0.15 Ca	1.84 ± 0.07 Abc	2.45 ± 0.28 Bb
	Frozen	3.47 ± 0.04 Cb	1.93 ± 0.07 Ac	2.29 ± 0.04 Bb
	Cooked	NA	1.50 ± 0.03 Aa	1.98 ± 0.12 Ba
	Cooked-Frozen	NA	1.71 ± 0.03 Ab	2.21 ± 0.02 Bb

* Average values ± standard deviations of three replicates (*n* = 3). Within each group and for each row, different capital letters (A,B,C) indicate significant differences (*p* < 0.05) among tissues. Within each group and for each column, different lowercase letters (a,b,c) indicate significant differences (*p* < 0.05) as a result of processing. ** Specimen sizes are as expressed in Table 1. *** NA: not analyzed.

**Table 7:** Determination of the flesh-lipid quality (FLQ) index * in different kinds of raw and processed tissues corresponding to different specimen sizes **.

Specimen Size	Raw or Processed Tissue	Tissue		
		Viscera	Mantle	Arm
Group I	Raw	58.32 ± 5.15 Ab	80.86 ± 6.25 Bbc	73.52 ± 1.44 Bb
	Frozen	48.63 ± 2.34 Aa	73.79 ± 2.44 Cb	66.86 ± 2.22 Ba
	Cooked	NA ***	83.39 ± 2.17 Bc	72.35 ± 3.18 Aab
	Cooked-Frozen	NA	65.18 ± 3.27 Ba	76.15 ± 3.16 Ab
Group II	Raw	64.99 ± 1.12 Ab	74.40 ± 2.09 Bb	99.16 ± 3.26 Cb
	Frozen	55.09 ± 2.35 Aa	80.70 ± 3.20 Bc	82.27 ± 4.08 Ba
	Cooked	NA	78.19 ± 2.10 Abc	86.92 ± 4.38 Ba
	Cooked-Frozen	NA	66.81 ± 3.43 Aa	82.08 ± 3.39 Ba
Group III	Raw	47.67 ± 3.51 Ab	76.21 ± 3.42 Bab	77.37 ± 3.21 Ba
	Frozen	56.25 ± 1.41 Aa	72.18 ± 4.41 Ba	81.59 ± 1.40 Cab
	Cooked	NA	77.09 ± 2.37 Aab	82.32 ± 1.17 Bab
	Cooked-Frozen	NA	79.53 ± 2.17 Ab	84.09 ± 1.12 Ab

* Average values± standard deviations of three replicates (*n* = 3). Within each group and for each row, different capital letters (A,B,C) indicate significant differences (*p* < 0.05) among tissues. Within each group and for each column, different lowercase letters (a,b,c) indicate significant differences (*p* < 0.05) as a result of processing. ** Specimen sizes are as expressed in Table 1. *** NA: not analyzed.

## Data Availability

The original contributions presented in the study are included in the article; further inquiries can be directed to the corresponding author.
